# Readiness of rural health facilities to provide immediate postpartum care in Uganda

**DOI:** 10.1186/s12913-023-09031-4

**Published:** 2023-01-10

**Authors:** Mariam Namutebi, Gorrette K. Nalwadda, Simon Kasasa, Patience A. Muwanguzi, Cynthia Kuteesa Ndikuno, Dan K. Kaye

**Affiliations:** 1 Department of Nursing, School of Health Sciences, College of Health Sciences, Makerere University, P.O Box 7072, Kampala, Uganda; 2 Department of Epidemiology and Biostatistics, School of Public Health, College of Health Sciences, Makerere University, Kampala, Uganda; 3 Department of Obstetrics and Gynecology, School of Medicine, College of Health Sciences, Makerere University, Kampala, Uganda

**Keywords:** Facility readiness, Postpartum care, Facility assessment

## Abstract

**Background:**

Nearly 60% of maternal and 45% of newborn deaths occur within 24 h after delivery. Immediate postpartum monitoring could avert death from preventable causes including postpartum hemorrhage, and eclampsia among mothers, and birth asphyxia, hypothermia, and sepsis for babies. We aimed at assessing facility readiness for the provision of postpartum care within the immediate postpartum period.

**Methods:**

A cross-sectional study involving 40 health facilities within the greater Mpigi region, Uganda, was done. An adapted health facility assessment tool was employed in data collection. Data were double-entered into Epi Data version 4.2 and analyzed using STATA version 13 and presented using descriptive statistics.

**Results:**

Facility readiness for the provision of postpartum care was low (median score 24% (IQR: 18.7, 26.7). Availability, and use of up-to-date, policies, guidelines and written clinical protocols for identifying, monitoring, and managing postpartum care were inconsistent across all levels of care. Lack of or non-functional equipment poses challenges for screening, diagnosing, and treating postnatal emergencies. Frequent stock-outs of essential drugs and supplies, particularly, hydralazine, antibiotics, oxygen, and blood products for transfusions were more common at health centers compared to hospitals. Inadequate human resources and sub-optimal supplies inhibit the proper functioning of health facilities and impact the quality of postpartum care. Overall, private not-for-profit health facilities had higher facility readiness scores.

**Conclusions:**

Our findings suggest sub-optimal rural health facility readiness to assess, monitor, and manage postpartum emergencies to reduce the risk of preventable maternal/newborn morbidity and mortality. Strengthening health system inputs and supply side factors could improve facility capacity to provide quality postpartum care.

## Introduction

There is a global average of 830 maternal deaths and 6700 newborn deaths daily from preventable causes with majority of these occurring in developing countries [1–3]. In Uganda, the maternal mortality ratio has steadily declined by approximately 1 % annually over the last 5 years and now stands at 375 deaths per 100,000 live births, while the neonatal mortality rate has stagnated at 27 per 1000 live birth since 2006 [2, 4]. More than 60% of maternal deaths and 45% of newborn deaths occur in the postpartum period (defined as the period beginning 1 hour after the delivery of the placenta and continuing until 6 weeks (42 days) after delivery [3, 5, 6]). The causes of these maternal deaths are known: postpartum hemorrhage, eclampsia, sepsis, and indirect causes [7]. Newborn deaths are commonly due to: birth asphyxia, prematurity, sepsis, tetanus and diarrhea [8, 9]. These can depend on delays at various levels as proposed by Thaddeus, including the delay in making the decision to seek care (type 1 delay), reaching the healthcare facility (type 2 delay), and receiving appropriate care (type 3 delay) [10]. Type 3 delays include factors affecting the speed with which effective care is provided once a woman reaches a healthcare facility, which can be affected by health facility readiness.

Facility readiness is a measure of how prepared health facilities are to provide health services and is measured by the availability of components required to provide services like adequate qualified and trained staff, guidelines on the provision of care, basic amenities, basic equipment, standard precautions, laboratory tests, and medicines and commodities [11, 12]. Facility readiness may not be a direct indicator of quality of care, however, it is a proxy measure of quality of care and how prepared the facility is to avail this care when it is needed [13].

High facility readiness is crucial for the achievement of the third Sustainable Development Goal (SDG) to reduce the maternal mortality ratios and neonatal mortality rates by 2030 [14, 15]. One of the indicators of the achievement of this SDG is the percentage of women who deliver their babies in health facilities under a skilled attendant. A lack of improvement in maternal and newborn outcomes has been noted, despite trends in increased proportions of facility-based deliveries, especially in low-income countries [16]. This may be due to a lack of corresponding changes in facility readiness and responsiveness to obstetric and newborn emergencies [17].

The World Health Organization (WHO) advocates that women and newborns receive care from motivated, skilled practitioners in a safe and dignified environment that regularly has the necessary equipment and drugs [12]. Lack of facility readiness to provide maternity services may result in many women seeking alternative care such as going to traditional birth attendants [18], which may put their health at risk. This not only jeopardizes the health of individuals, but also erodes trust and puts entire health systems and populations at risk [19]. In contrast, facilities with high readiness scores contribute to high-quality health systems that seek to provide integrated services, strive to promote equitable services, earn the trust of the people they serve through positive experiences of care and deliver better quality care [20].

Ending preventable maternal deaths continues to be one of the most important goals internationally with a target of less than 70 maternal deaths per 100,000 live births by the year 2030 [14]. Health facility readiness can contribute to the achievement of this goal by ensuring preparedness to handle postpartum emergencies among women and their newborns. However, there is limited information regarding the facility readiness to provide postpartum care in Uganda, save for the information about the high patient to health worker ratio [17]. Therefore, this study aimed at assessing the level of facility readiness to provide care to mothers, and newborns in the immediate postpartum period (delivery of the baby to the end of the first 24 hours or discharge from the health facility (whichever comes first)) in the greater Mpigi region, Uganda.

## Methods

### Study design

This was a cross sectional study that employed quantitative methods of data collection using a facility assessment tool. This study design was adopted because we sought to collect data at a single point in time to assess how prepared the facilities were to provide care to women and babies within the immediate postpartum period.

### Study setting and population

The study setting was in the greater Mpigi region found in central Uganda, which consists of three districts namely: Butambala, Gomba, and Mpigi. This region was chosen because it is centrally located and it has health facilities in both the peri-urban and rural settings, which serve a wide population. The health system in Uganda has several tiers including: health centers I, II, III, IV; general hospitals; regional referral hospitals; national referral hospitals; and national specialized hospitals. A health center I is located at the village level and provides basic home-based care and health promotion. The care at this level is provided by the village health teams comprised of volunteers in the community. Health center IIs are located at the parish level and provide outpatient curative services and outreach for health promotion. At the sub-county level, we have health center IIIs, which provide all the above and inpatient medical, antenatal and maternity care. Next is the health center IV at the health sub-district, where emergency surgical and maternity care, blood transfusion and laboratory services are available, together with the health promotion and outpatient curative care. Then we have the general hospital at the district level that provides all the care and services provided by the health center IVs, in addition to general surgical care, mental health care, dental care and some specialized services like physiotherapy. At the regional referral hospitals, clients can access specialized medical services. These also offer training for different cadres of health workers. There are 9 national-level hospitals, of which 5 are specialized. None of these national referral or specialized hospitals were included in this study.

The target population for this study were all the health facilities in our study area that provide intrapartum care services and health facility based postpartum care which include: hospitals, health center IVs and health center IIIs according to the current Ministry of Health (MOH) policy guidelines for health care services [14, 21].

### Eligibility criteria

The facilities in the greater Mpigi region that were eligible to participate in this study were those that conducted deliveries and had been providing the service for at least 6 months at the time of the data collection. The three district health officers and all included facility administrators provided administrative clearance for the study to be conducted at their health facilities. Thereafter, permission was sought from the maternity ward in-charges to conduct the study on their units. Informed consent was sought from all the midwives and mothers who participated in the study.

### Sample size and sampling

The sample size of 40 health facilities was calculated using the simplified formula by Yamane [22] at a confidence interval of 95%, error of 5%, and a total population of 44 health facilities in the greater Mpigi region that provided delivery services based on the national health facilities inventory [21]. There were two hospitals, three health center IVs and 39 health center IIIs in the official list of registered health facilities in the three districts [21]. Out of 44 health facilities, 40 health facilities were assessed in this study. All the hospitals and three health center IVs in the three districts plus all the health center IIIs in Butambala and Gomba were purposively included in the study because they were few. Health center IIIs within Mpigi district were recruited consecutively until all the required sample (for Mpigi district) of 17 health center IIIs was reached.

### Data collection tools

The data were collected using a facility assessment tool that has been adapted from the Ministry of Health’s original result-based financing (RBF) assessment and the SARA facility assessment tools [23]. The MOH tool’s sections were retained but adjusted to focus on postpartum care provision. The tool has eight sections including; presence of a written policy on hospital stay after delivery (scored as 5 or 0), availability of a checklist for routine monitoring of women post-delivery (scored as (15 to 0), presence of a clean, private, dedicated area for postpartum care (scored as 5 or 0). presence of tracer medicines and commodities for basic obstetric care (oxytocin, misoprostol, magnesium sulphate, amoxicillin, metronidazole, vitamin K, tetracycline chlorhexidine gel, normal saline, ringers lactate, 50% dextrose, surgical gloves, examination gloves, sutures (vicryl), gauze, alcohol, hibitane solution and surgical blades) (scored as 5 or 0), availability of a checklist for counselling postpartum women (scored as 15 to 0), availability of tracer equipment for postpartum care provision (blood pressure machines, thermometer. tape measures, glucometer, pulse oximeters) (scored as 15 or 0). presence of a functional transport system (scored as 5 or 0), presence of a viable client information and educational program (scored as 10 or 0), Each section was scored separately and an overall score for the facility was computed with a maximum score of 70 and a minimum score of zero. The scores were then transformed into percentage scores. The median scores per district, facility level, and facility type were computed. The tool was checked for content validity by DKK and GKN, then pilot tested by assessing two senior midwives working at a referral hospital in Kampala. Changes were made to the tool as suggested by the midwives and the two coauthors. During the training of the research assistant, care was taken to minimize inter-rater variability to increase the reliability of the scores. Whenever possible, the two assessors both scored the facility visited and compared their scores before agreeing on the final facility score.

The dependent variable for this study was the facility readiness for the provision of postpartum care, which was the median score for all the facilities based on the facility readiness assessment scores. The independent variables included: facility level, ownership, number of midwives, district, average number of deliveries per month, and number of postnatal beds.

### Data collection procedures

Data were collected over a period of 4 months between August and December 2020. After verifying the functionality of the facilities identified in the national health facility inventory [21] with each district health office, each facility in-charge or medical superintendent was contacted by telephone or email and briefed about the study before the facility was visited. Those that responded positively were scheduled for a visit and the in-charge was informed of the possible date for the visit. On arrival at the health facility, administrative clearance was sought from the in-charge who introduced the study team to the midwife on duty or the maternity in-charge. The team then explained what they needed to do and proceeded to complete the assessment with the help of either the midwife or the maternity in-charge. Those facilities where administrative clearance was not obtained were excluded from the study.

Various items were assessed in the facility readiness assessment tool including: length of hospital stay following a normal delivery; routine monitoring of postpartum care; availability of space; tracer medicines; commodities; equipment; sundries; a functional transport system; and a health education program for the postpartum women and their care givers. Completion of the assessment tool included verification of the presence or absence of the various equipment, drugs, postnatal charts, MOH guidelines for postpartum care, standard operating procedures for the provision of postpartum care, visual reminders for midwives / checklists for care provision, discharge guidelines, health education guides (models, flip charts, posters, time table, and other teaching aids), brochures for information provision, examination room / couch, cleaning materials and cleaning schedule for the postnatal unit. For sections where there was observation of care being provided, the team requested to be present while care was being provided to a postpartum mother so as to observe the care provided to the client. No identifying information regarding the clients was collected, as the researchers were only interested in observing what the midwife did in providing care. The clients were informed about the study and requested for consent to be observed while receiving care. In facilities where there were no clients found at the time of the visit, the midwife was asked to describe what she does for each client from the time of delivery of the baby to the time of discharge, and this was assessed against the postpartum care guidelines for both the mother and newborn. The midwife was then asked to avail the team with policy guidelines and checklists that were being used for the provision of care and health education. She also took the team to the ward, store and laboratory where they checked for the availability of drugs, sundries (IV giving sets, blood giving sets, cotton, gauze, examination gloves, surgical gloves, and gynecological gloves), equipment, blood products, and laboratory equipment. Regarding the postpartum counseling done and documented by the midwife. We interviewed one midwife at each health center III and health center IV. At the hospitals, we interviewed and observed 3 midwives providing in-facility postpartum care but only one midwife who was the maternity in-charge assisted the researchers in ascertaining the presence of the required documents, drugs and equipment. The number of midwives assessed was based on the guidance from the tool and the availability of the midwives/ patients at the health facility at the time of the facility assessment. The team spent at least 1 day at each facility depending on the availability of the facility in-charge, midwife and the postpartum clients. Each filled assessment tool was reviewed for completeness before leaving the facility and any missed observations were completed before departure from the facility.

### Data management and analysis

Data were double entered into Epi data version 3.1, cleaned, and exported to Stata version 14.2 for analysis [24, 25]. Univariate analysis was performed for health facility characteristics and presented as frequencies and percentages. All facilities that scored 80% and above were considered to be prepared to provide postpartum care while those that scored below the 80% were considered not ready to provide postpartum care. The facility readiness score was also reported in a table reflecting minimum and maximum scores and the median per district because the scores were skewed to the left. Higher scores denoted that a facility was better prepared to provide postpartum care.

## Results

The greater Mpigi region has three districts from which the facilities were selected. The majority of health facilities were government owned and half of them 20/40 were within Mpigi district (Table 1). Among these, two were hospitals, three were health center IVs and 35 were HC IIIs. The health facilities had an average of three midwives per facility (range 1-13) and the number of deliveries per facility ranged from 1 to 450 per month. The midwives provided the following services: antenatal care, intrapartum and postpartum care, immunization, family planning, postnatal care, maternal-child health care outreaches and, general outpatient care services [26].

**Table 1:** Characteristic of the health facilities assessed within the greater Mpigi region

Variable	Total (***N*** = 40) n (%)	HCIII (***N*** = 35) n (%)	HCIV (***N*** = 3) n (%)	Hospital (***N*** = 2) n (%)
**District**				
Mpigi	20 (50.0)	17 (48.6)	2 (66.7)	1 (50.0)
Gomba	11 (27.5)	10 (28.6)	1 (33.3)	0 (0.0)
Butambala	9 (22.5)	8 (22.9)	0 (0.0)	1 (50.0)
**Health facility type**				
Government	27 (67.5)	24 (68.6)	2 (66.7)	1 (50.0)
PNFP	8 (20.0)	7 (20.0)	0 (0.0)	1 (50.0)
PFP	5 (12.5)	4 (11.4)	1 (33.3)	0 (0.0)
**Number of Midwives**				
**1 to 2 midwives**	20 (50.0)	20 (57.14)	–	–
**3 to 4 midwives**	11 (27.5)	11 (31.43)	–	–
**5 to 14 midwives**	9 (22.5)	4 (11.43)	3 (100.0)	2 (100.0)
**Number of deliveries**				
1 to 20 deliveries	14 (37.5)	13 (37.1)	1 (33.3)	–
21 to 100 deliveries	22 (52.5)	21 (60,0)	1 (33.3)	–
More than 100 deliveries	4 (10.0)	1 (2.9)	1 (33.3)	2 (100.0)
**Number of postnatal beds**				
No beds available	5 (12.5)	5 (14.29)	–	–
1 to 4 beds	23 (57.5)	22 (62.86)	1 (33.3)	–
5 to 9 beds	9 (22.5)	8 (22.85)	1 (33.3)	–
10 or more beds	3 (7.5)	–	1 (33.3)	2 (100.0)

Of the 40 facilities, 5/40 (12.5%) had no dedicated postpartum beds available while the majority had 1-to-4 beds (Table 1).

### Health facility readiness in the selected facilities

#### Policy on length of hospital stay after a normal delivery

All the facilities scored no points for this section (Table 2), because none had a written policy on hospital stay, while others had insufficient space to keep postpartum mothers for a long time.

**Table 2:** Distribution of points scored in the different sections of the facility assessment checklist by the different facilities

Section	Requirements to attain the points	Points	HCIII n (%)	HCIV n (%)	Hospital n (%)	Total number of Health facilities N (%)
1	There is a written policy on facility stay and mothers stay at the facility for at least 24 h following a normal delivery (Score 5 or 0)	0	35 (100.0)	3 (100.0)	2 (100.0)	40 (100.0)
		5	–	–	–	–
2	Facility has a checklist for monitoring the mother (day after delivery and examining her at the time of discharge. There is a written policy on postpartum visits and women are informed about when to return. (Score 0 to15)	0	–	1 (33.3)	–	1 (2.5)
		3	1 (2.86)	–	1 (50.0)	3 (5.0)
		5	19 (54.3)	–	–	19 (47.5)
		7	1 (2.9)	–	–	1 (2.5)
		8	5 (14.3)	–	1 (50.0)	6 (15.0)
		10	8 (22.9)	2 (66.7)	–	11 (25.5)
		12	1 (2.9)	–	–	1 (2.5)
3	There is a separate area for the provision of postpartum care with beds and a clean private environment (both visual and sound). The place has accessible and clean wash rooms and toilets. (Mopped floors, wall and ceiling free of cobwebs and in a good state of repair). (Score 0 or 5)	0	30 (85.7)	2 (66.7)	1 (50.0)	33 (82.5)
		5	5 (14.3)	1 (33.3)	1 (50.0)	7 (17.5)
4	Observation of availability on day of assessment and verification whether stock card is up to date and quantities tally with physical stock. (Score 5 or 0)	0	30 (85.7)	2 (66.7)	2 (100.0)	34 (85.0)
		5	5 (14.3)	1 (33.3)	–	6 (15.0)
5	All postpartum women receive written and verbal information/ counseling before discharge (Score 0 to 15)	0	1 (2.9)	–	1 (50.0)	2 (5.0)
		2	–	1 (33.3)	–	1 (2.5)
		3	16 (45.7)	–	–	16 (40.0)
		4	1 (2.9)	–	–	1 (2.5)
		5	17 (48.6)	2 (66.7)	1 (50.0)	20 (50.0)
6	Observation of the equipment in the postnatal ward and check functionality (Score 15 or 0)	0	34 (97.1)	2 (66.7)	–	36 (90.0)
		15	1 (2.9)	1 (33.3)	2 (100.0)	4 (10.0)
7	Observation of vehicles and vehicle logs (Score 5 or 0)	0	3 (8.57)	–	–	3 (7.5)
		5	32 (91.4)	3 (100.0)	2 (100.0)	37 (92.5)
8	Observation of IEC materials in each service delivery area (Score 10 or 0)	0	34 (97.1)	2 (66.7)	2 (100.0)	38 (95.0)
		10	1 (2.9)	1 (33.3)	–	2 (5.0)

The length of hospital stay after delivery ranged from 4 to 48 h. At some facilities, mothers were discharged before the recommended 24 h by reason of their condition or the desire of the mother to be discharged.

#### Routine monitoring of postpartum women and their babies

For this section, 19/40 (47.5%) of the facilities scored 5 out of 15 points (Table 2) because they lacked written policies on postpartum monitoring and pre-discharge care or the guidelines were not being followed. Some mothers were being monitored for 30-to-60 min and then not seen till discharge.

#### Availability of a dedicated functional space or ward for postpartum mothers

The majority of the facilities 33/40(82.5%) did not score any points in this section since they had inadequate space, no privacy and insufficient beds for their clients (Table 2). For the newly built or upgraded facilities, the postpartum area often comprised of an empty space with no beds or mattresses. Some facilities also had un-sanitary latrines or toilets which were not in use or closed, whereas there were facilities with leaking roofs and falling ceilings too. Only three facilities had partitioned the postnatal spaces with curtains and another two had improvised separate rooms to ensure privacy for their clients. Where the number of deliveries were high, it was common to find mothers with their babies sleeping on the floor due to lack of beds.

#### Availability of tracer medicines and commodities

Regrettably, 34/40 (85.0%) of the facilities scored 0 out of 5 points (Table 2) because chlorhexidine gel, hydralazine, examination and gynecological gloves were out of stock in 17/40 (42.5%), 21/40 (52.5%), 7/40 (17.5%) and 11/40 (27.5%) facilities respectively as shown in Table 3 below. We also noted that health center IIIs had side laboratories to provide basic laboratory screening services only. Provisions for laboratory services, including blood transfusion services, were available at health center IVs and hospitals as stipulated in the MOH guidelines.

**Table 3:** Number of facilities having specified drugs or supplies at the time of assessment

Drug/ supply	Frequency (***N*** = 40)	Percentage (%)
**Anti-hypertensives**	13	32.5
**Antibiotics**^**a**^	15	37.5
**Chlorhexidine gel**	23	57.5
**Diazepam**	24	60.0
**Gynecological gloves**	29	72.5
**Modern family planning**^**b**^	29	72.5
**Examination gloves**	33	82.5
**Magnesium sulphate**	37	92.5
**Oxytocin**	40	100
**IV giving sets**	38	92.5

^a^These included; Amoxyl, Ampicillin, Gentamycin and Ceftriaxone, and metronidazole

^b^These included; Hormonal methods of Family planning like pills, depo provera, implanon, Jadelle, Barrier methods like the Male and female condoms and others such as the IUDs

#### Postpartum counselling

The maximum score attained was 5 out of 15 points which was achieved by 20/40 (50.0%) of the facilities (Table 2). The reasons for the scores were: there were no guides and no written record of the information given. At some facilities, the women’s notes were written in books that were given to them at discharge making it hard to verify what was documented in them. This hindered our scoring of this section as we then had to assess the midwives’ knowledge of what information is provided to the mother rather than their practices.

#### Presence/ functionality of tracer equipment

Half of the health facilities (20/40) scored 5 out of 15 points under this section (Table 2). Hospitals and health center IVs were better equipped than health center IIIs. Nearly all the facilities had examination couches, sutures, blood pressure machines and newborn resuscitation equipment (resuscitation table, sucker, ambu bags and face masks). However, some health facilities lacked adult resuscitation equipment (suction machine, ambu bags and face masks, resuscitation tray) (24/40, 60%), examination lights (17/40, 42.5%), sterilization gear which was either an autoclave or a stove and saucepans for boiling the equipment (19/40, 22.5%) and oxygen (32/40, 77.5%). Details for available equipment are shown in Table 4.

**Table 4:** Number of health facilities with selected essential equipment and supplies

Variable	Frequency /percentage
**Equipment**	
Adult resuscitation equipment (ambu bag, face mask, and suction)	16 (40%)
Newborn resuscitation equipment (ambu bag, sucker, and face mask)	36 (90%)
Examination couch	39 (97.5%)
Length meter	32 (80%)
Thermometer	36 (90%)
B.P machine	35 (87.5%)
Examination Light	23 (57.5%)
Baby weighing scale	34 (85%)
Sterilization equipment (presence of autoclave or stoves for boiling equipment)	31 (77.5%)
Speculums	38 (95%)
**Supplies**	
Sutures	36 (90%)
Oxygen	8 (20%)

#### Access to transport

Nearly all facilities 37/40 (92.5) scored 5 because they had access to a vehicle to transport clients in need of referral (Table 2). The hospitals and health center IVs had functional ambulances that were used by most facilities in the region to transport women and babies needing referral. However, the availability of the vehicles was dependent on the patients paying a fee for fueling the cars in order to be transported. This was noted to hinder quick referral of patients in some cases,

#### Availability of a functional health education program

Majority of the health facilities 38/40(95.0) scored 0 under this assessment category (Table 2) because they did not have health education materials available, especially those with regard to modern family planning methods. We assessed the provision and availability of all methods of family planning but especially, the postpartum IUD and implants (Table 3). For the provision of family planning services, we assessed the availability and access for the postpartum mother before discharge. Only one facility had a midwife that had been trained in the provision of postpartum IUDs but she was not providing the services at her facility. In most facilities the family planning services were provided by a different team or on specific days making it hard to access if the woman desired to leave the facility with a family planning method at the time of discharge, post-delivery. There were facilities with no health education timetables, while others had books that had not been updated for months. Facility readiness assessment for this category are further detailed in Table 5.

**Table 5:** Facility readiness for provision of immediate postpartum care tool

Dimension	Available points	Where to find information needed to evaluate standard	Requirements to attain the points	Operational definition	Points assigned
1.	5 or 0	Ask to see the written policy on facility stay after a normal delivery. Observe 2-5 women from the time of delivery up to discharge. Indicate if the policy was implemented in their care.	There is a written policy on facility stay and mothers stay at the facility for at least 24 h following a normal delivery.	Healthy mothers stay at the facility for at least 24 h following a normal delivery	5 Points awarded if policy exists and is being implemented, otherwise facility scores 0 points
2.	15 to 0	Ask to see a checklist for routine monitoring of the woman post-delivery and at the time of discharge. Verify that the checklist is used in monitoring the mothers post-delivery and before discharge. Verify that the mother is examined prior to discharge and knows when to return. Check for a copy of the current MOH guidelines for postpartum care on the ward.	Facility has a checklist for monitoring the mother after delivery and examining her at the time of discharge. There is a written policy on postpartum visits and women are informed about when to return. (day 6 and at 6 weeks).	All postpartum mothers are monitored according to the guidelines and examined before discharge.	Assign score of the checklist 3, policy 2, examination 5, MOH guidelines 2, mother’s knowledge of when to come back 3.
3.	5 or 0	Ask to see the postpartum area. Observe and verify privacy/ cleanliness in the area. Check to see the postnatal beds.	There is a separate area for the provision of postpartum care with beds and a clean private environment (provides both visual and sound privacy). The place has accessible and clean wash rooms and toilets. (mopped floors, wall and ceilings free of cobwebs and in a good state of repair)	There is sufficient space or a ward assigned to the postpartum mothers.	If all the items are available 5 marks otherwise 0
4.	5or 0	Does the facility have tracer medicines and commodities for Basic Obstetric Care	Observation of availability on day of assessment and verification whether Stock card is up to date and quantities tally with physical stock	Check that the following are present; Uterotonics (Oxytocin, misoprostol) Magnesium Sulphate Caps amoxicillin Chlorhexidine IV diazepam IV hydralazine Vitamin K Tetracycline eye ointment Rectal diazepam Ampicillin inj Gentamicin inj Ceftriaxone inj Examination gloves Surgical gloves Gynecological gloves Normal saline IV cannulas IV giving sets Cotton gauze Blood giving sets Blood in the blood bank/ fridge Postpartum IUDs and other contraceptive methods	Score 5 if the facility has tracer medicines and commodities for Basic Obstetric Care and the stock cards are up to date.
5.	15 to 0	Ask to see checklist for postpartum counseling. Observe midwives as they counsel 3 women at discharge. If there is no patient use a mannequin. Note that the staff includes all the topics and the advice given to the mother is documented.	All postpartum women receive written and verbal information/ counseling before discharge	Staff counsel mothers before discharge. Checklist for postpartum counseling includes; self-care, hygiene, nutrition, family planning, breast care, perineal care, danger signs for mother and baby.	Assign score of; presence of guide 3, correct guide content 3, correct demonstration 3, recording of advice 2, written information given 5.
6.	15 or 0	Does the facility have tracer equipment for postnatal ward?	Observation of the equipment in the postnatal ward and check functionality	Thermometer B.P machine weighing scale (adult and baby) Stethoscope Speculums Examination light Sterilization Equipment Examination bed Suturing kit (needle holder, Stitch scissors forceps, and sterile pack) Suction machine Ambu bags (adult and newborn) Face mask (adult and newborn) Airway (different sizes). Sutures Length meter Oxygen cylinder and /or concentrator Patient monitor Pulse oximeter	Give a score of 15 if all equipment is present and in good working condition.
7.	5 or 0	Does the health facility have a functional transport system?	Observation Of vehicles and vehicle logs	a) The facility has a functional motorized means of transport to cater for emergency transport of patients and clients. b) A functional ambulance or other vehicle for emergency transportation for clients that is stationed at this facility or operates from this facility OR Have access to an ambulance or other vehicle for emergency transport for clients that is stationed at another facility or that operates from another facility	Give a score of 5 if they have a functional transport system with up to date vehicle logs or access to a transport system that is reliable. Otherwise score as 0.
8.	10 or 0	Does the health facility have a functional health education program in the RMNCAH?	Observation of IEC materials in each service delivery area a) Service providers use one or more of the following materials during client counseling/ education sessions: ✓ FP methods ✓ anatomical models (Pelvis & penis dildo) ✓ FP flipcharts or Cue cards ✓ sample foods or demonstration garden. ✓ Observation of the posters at RMNCAH Clinics ✓ Observe time table for health education. ✓ Observe Health Education session in the RMNCAH clinic. Topics observed can include any of the following. • HIV/AIDS/PMTCT • Birth and emergency preparedness plan • Maternal and Newborn danger signs • Maternal Nutrition • Infant and Young Child feeding • Early Infant Diagnosis of HIV • Immunization • Family planning	FP methods and brochures Brochure on maternal and newborn danger signs F/P flip charts	Give a score of 10 if the facility has all the indicators for a functional health education program. Otherwise give a score of zero.
	Overall score				

This facility assessment tool has been adapted from the ministry of health facility assessment tool and is designed to assess the facility readiness to provide facility based postpartum care. It has eight sections and a maximum score of 75points. Follow the point awarding system embedded in the tool when assessing each facility

The overall median score for health facility readiness was 24% (IQR: 18.7, 26.7). The minimum score was 9.3% and the maximum score was 60% (Table 6). We had set our cut-off for facility readiness to provide postpartum care at 80%, based on the cut-off for the MOH facility assessments for the RBF program and other studies done in Malawi and Uganda, but no health facility scored above 80% in this study [27, 28].

**Table 6:** Health facility readiness by district, facility level and ownership among facilities assessed within greater Mpigi region

Variable	Median of Score (IQR)	Minimum score (%)	Maximum score (%)
**District**			
Mpigi	23.4 (17.3, 26.7)	9.3	60.0
Gomba	26.7 (21.3, 33.3)	10.7	46.7
Butambala	20 (17.3, 26.7)	17.3	37.3
**Facility level**			
HC III	22.7 (17.3, 26.7)	10.7	46.7
HC IV	40 (9.3, 60.0)	9.3	60
Hospital	40.7 (30.7, 50.7)	30.7	50.7
**Facility type**			
Government	21.3 (17.3, 26.7)	9.3	40
Private not for profit	26.7 (20.0, 30.0)	20.0	50.7
Private for profit	24 (22.7, 46.7)	21.3	60

When the health facility readiness score was analyzed for the facility level and facility type. Hospitals were found to have a higher overall median score (40.7%; IQR: 30.7, 50.7), while health center IIIs had the lowest median score (22.7%; IQR: 17.3, 26.7). The private not for-profit facilities also had a higher median score (26.7%; IQR: 20.0, 30.0) compared to government-owned and private for profit facilities which had median scores of 21.3% (IQR: 17.3, 26.7) and 24.0% (IQR: 22.7, 46.7) respectively (Table 6).

## Discussion

This study aimed at assessing the level of facility readiness for the provision of postpartum care within the greater Mpigi region in Uganda. The overall scores for facility readiness ranged from 9.3 to 60% in this study. These scores were very low compared to the cutoff point (80%) for the MOH facility assessment tool that is used for facility assessments in the RBF program being implemented in the region. RBF is a world bank funded government initiative in Uganda that is aimed at improving the maternal, newborn, child and adolescent health (MNCAH) services in the country through offering financial incentives to health facilities that achieve the desired performance for defined indicators for the MNCAH outcomes. It has been rolled out progressively in the country over the last 5 years and had just been rolled out in the greater Mpigi region at the start of the data collection for this study [29]. Some of the indicators assessed for by the RBF team were overlapping with the ones assessed in this study such as presence of guidelines and their implementation in care provision. They also assessed for adequacy of the infrastructure, presence of equipment, sundries, and drugs. The aim of the assessments is to scale up the adoption of the WHO standards for MNCAH care within the whole country and as such improve the quality of care [29, 30]. Though this could have affected the facility scores for facilities that were participating in the RBF project, the overall effect of this on the findings could not be assessed in this study. However, we noted that since the project was just being rolled out in the region, many facilities were undergoing changes in the implementation of the care provision.

The study shows that the health facilities had not taken up the policies and guidelines that would improve facility readiness for emergency obstetric care. The MOH issued new guidelines in 2016 for the provision of postpartum care based on the WHO recommendations [5, 26], however these guidelines were not being implemented with regard to length of hospital stay and patient care after delivery. This is similar to findings from other studies done in Uganda and other low and middle-income countries [6, 31, 32] which found that most midwives were basing their practice on their pre-qualification training and not on the current MOH guidelines. Studies done in Tanzania, Malawi and other countries also showed that many health workers did not know or provide care according to the stipulated postpartum care guidelines [27, 31, 33].

Though the current guidelines were introduced over 5 years ago, the use of checklists for postpartum monitoring was reported to have been recently introduced in some facilities to meet the requirements by supervisors for the RBF activities in the region at the time of the study. As such, in some facilities where protocols had been enacted, the postpartum care documentation was noted to be incomplete or filled retrospectively. This is not surprising as it has been found that the translation of policies and guidelines into practice is slow and is usually dependent on the implementing institution’s ability to adapt and own the proposed guidelines [34]. This practice could also be due to the midwives’ current work load or the perceived importance of the documentation of care [35, 36]. In most facilities we visited, the midwives were alone on duty on the day of the facility assessment, hence they could have been overwhelmed by their duties [35].

Considering that this study was conducted at the start of the financial quarter for government facilities, many had the necessary equipment, drugs, and sundries. However, there were still several facilities that had shortages for essential drugs, and sundries (IV giving sets, cannulas, cotton, gauze, surgical gloves, examination gloves, and gynecological gloves). This is an impediment to facility readiness and is a hindrance to the effective provision of care to the clients, and could be a source of stress for the health workers as they provide care [32]. This finding is in agreement with studies done within other low income countries where many facilities lacked essential equipment and drugs used in the provision of health care services [27, 32]. This is a long-standing challenge for these countries, signifying failure of the health systems to consistently ensure that facilities have the required drugs, equipment and supplies at all times. Furthermore, hospitals and health center IVs were found to be better equipped than the health center IIIs yet the health center IIIs are the first line of access to postpartum care and do serve large populations especially in the rural areas. Although this is not an unexpected finding, it has been noted to be a hindrance in meeting the population’s needs and the meeting of the global goals of reduction in maternal and newborn mortality through the provision of accessible and effective care to all populations [37–40]. Health center IIIs need to be equipped with life-saving equipment to enable them to provide timely basic emergency care (especially, giving first line care like resuscitation, intravenous fluids, anti-hypertensive drugs, uterotonics, and intravenous antibiotics before referral) to their clients. Most of whom sometimes find themselves miles and hours away from the nearest hospital. The equipping of these facilities could reduce the time lag between diagnosis and intervention which may be critical in the life of the mother and her newborn [33, 40, 41].

It is important that healthcare providers are able to work in a well-functioning health system where good quality care can be provided. Adegoke et al. [42] described a well-functioning health system as one that provides an “enabling environment” that includes sufficient human and financial resources, essential drugs, and the necessary equipment [29]. This has been emphasized by the assessments and the WHO’s road map to improving maternal and newborn health services [31, 43]. Studies have shown that women may determine where to deliver from based on the setting, organization and available resources at the facility [32, 44]. These resources may include the environment, privacy and amenities like electricity and water. This, plus the clients’ perception of the facility’s preparedness to handle emergencies, may be one motivation for choice of which facilities they seek care from. This may result in some facilities being overcrowded while others have very few clients which may hinder effective care provision and affect the patients’ experience of the care.

The findings of low facility readiness have implications for the quality of care provided. For example, influencing patients’ experience of care, which largely depends on the outcomes of the care and the relationship between the client and the health workers. A facility that is not ready to provide care will likely produce delays in accessing care and fail to provide services, which, coupled with health workers that are frustrated because of the system constraints in care provision, is likely to result in a negative or poor experience of care for the client [45, 46]. One of the implications for the lack of facility policies and guidelines noted in this study was that length of hospital stay after a vaginal delivery was varied from 4 to 48 h at the different facilities. The shortened hospital stays and the lack of proper assessment and health education at the time of discharge may predispose women and their newborns to the risk of readmission due to complications and possible death in the community [47–49]. Only one facility stated that their postpartum clients were expected to stay for at least 48 h before discharge and this could have been related to the patient numbers and the availability of space and beds for the patients. A study done in Tanzania, showed that both patients and health care workers are aware of how lack of beds and space at the facility has made short facility stays after delivery a norm [50].

We note that, availability of facilities for patient care may not necessarily mean that these could be easily accessed by patients, further limiting validity of the facility readiness assessment tool. In addition, it does not mean that the guidelines or equipment are used for decision-making for patient care. For instance, although there were ambulances available at the government and private not-for-profit facilities (hospital and health center IVs), access to them by patients coming from lower health facilities was limited due to the fact that the patients needed to contribute funds for buying fuel before they were transported. These findings are similar to those of a study done in Uganda assessing the relationship between facility readiness and its effect on malaria outcomes, which found that availability of emergency transportation at health center III and II level was only 5.2% [37]. A study done in Northern Uganda found that lack of access to emergency transport led to delays and to referred patients being unaccompanied to the referral unit [41]. This greatly reduces the effectiveness of the referral system.

The study also noted a lack of integration of services, which affects facility readiness. For instance, more than half of the facilities (72.3%) that were assessed provided family planning services to their clients (Table 3). However, there was no integration of services (that is, the services were provided at different service points and by different health workers from those who provided intrapartum or postpartum care). This meant that the women needed to go to the antenatal clinic or come on a particular week day to the outpatient clinic to access the services hence immediate postpartum family planning services were not routinely provided to the women at the facilities. This constitutes a missed opportunity for providing family planning services immediately after delivery rather than at 6 weeks. Given the health-seeking behavior of most women in rural areas and the 28% unmet need for family planning in Uganda, this needs to be addressed to increase the clients’ access to the services [51]. The integration of family planning provision in HIV care has been noted to improve service delivery and the patients’ utilization of the services [52]. The WHO advocates for increased integration of care provision to increase service uptake, reduce fragmentation of the service provision, and meet the clients’ needs, and preferences [53]. This infers that the services are designed to respond to the needs of the women and that their preferences are considered as important. A study done in Nepal found that increase in external supervision would increase the facility scores for facility readiness for family planning integration in maternal, and child health care [54]. However, our study did not assess the frequency of supervision of the midwives within the study area.

Our study used a performance tool for the assessment of the facility readiness with a scoring criteria based on the all or no point score system for six of the eight domains. This could have led to some facilities scoring lower than they would have if the scoring criteria was cumulative or based on a scale. Hence the results of this study do not fully reflect which specific items under each indicator needs to be improved. Such composite soring criteria are usually used where one desires to compute the scores easily and also be able to compare the performance of different facilities. Critical review of the scores may be needed to define clearly what distinguishes the high performing facilities from the low performing ones as recommended by the article on composite measures in assessment of performance in health care [55].

### Strengths and limitations

This study was conducted in three districts and included different facility levels in rural and peri-urban settings and focused on care from the delivery of the baby to the time of discharge for women having normal deliveries. We used an objective method of assessment, hence the findings of our study are verifiable. We used a tool that was adopted from the RBF assessment tool because, at the time of conceptualization of the study, we noted that most facility assessment tools like the SARA were very broad and lacked some of the variables we were interested in assessing. As noted earlier, the scoring criteria for the tool was performance based, with an all or nothing composite scoring system for six of its eight categories. This could have been the reason for the overall low scores seen in this study. The low facility scores do not mean that the facilities were unprepared to provide any postpartum care but that they had gaps in some areas that could hinder their postpartum care provision. Secondly, concerning the signal function assessment for readiness to provide emergency obstetric care, we did assess the functions that spoke to immediate postpartum care only hence lack some of the aspects assessed by the SARA [56]. Thirdly, this was a cross-sectional study hence the findings speak to the time when the data was collected. In many facilities it was hard to access patients (most patients were discharged early in the morning) and the patients’ books where information about counselling and treatment provided were recorded, hence it was hard to verify which information was provided to the clients. To mitigate this, we requested the midwives to describe the way they monitored their clients, and enact health education sessions for their clients in order to complete the scoring for the facility. The authors recognize that this difference in approach of scoring the facilities may have introduced bias in the scores, because having the ability to describe what is supposed to be done is not necessarily equal to practice. The midwives could have changed their information provision to make it better or could have performed worse than they usually do due to anxiety. Finally, we considered the structural aspects of the provision of postpartum care only in this study, yet facility readiness should include assessment of the human resource readiness, and presence of functional laboratories [11, 33].

## Conclusions

The level of facility readiness for the provision of immediate and early postpartum care was found to be low. Since more than 40% of all maternal and newborn deaths occur on the day of delivery, there is a need to work at improving the facility readiness for postpartum care to reduce the maternal and newborn mortality rates in our setting. Development of hospital policies/ guidelines on postpartum care provision, improvements in infrastructure, distribution of the policies, practice guidelines and emergency transportation could improve the facility readiness for postpartum care provision.

## Definition of terms

1. **Immediate postpartum period:** In this study, this is the time from the delivery of a baby to the time of discharge from hospital or 24 h after the delivery of the baby depending on what precedes the other.
2. **Facility readiness:**
  This is the measure of the degree to which health facilities in the greater Mpigi region are prepared to provide postpartum care to their clients based on the availability of equipment, drugs, human resources, space, policies and standard operating procedures for postpartum care service provision. This was assessed using a facility readiness tool (Table 5) which has a maximum score of 70. Facilities with a score of 60 (80%) and above were considered to be prepared for the provision of postpartum care.
3. **Length of hospital stay:** For this study, the length of hospital stay was measured from the time of delivery of the baby to the time of discharge following an uncomplicated vaginal delivery.
4. **Postpartum care:** This is the care given to a woman and her baby from the time of the birth of the baby to 6 weeks after delivery. In this study we focused on the care provided in the first 24 h after the birth of the baby or till the time the mother and baby are discharged from the hospital whichever precedes the other.
5. **Postpartum assessments:** Postpartum assessment meant the examination of the mother baby pair post-delivery while monitoring focused on the sequential recommended monitoring of the mother and baby based on the Ministry of Health guidelines at the time (immediately after delivery, every 15 min for the first 2 hours then every hour till the fourth hour after delivery, then at 6 hours and four hourly till discharge if all is well

## Data Availability

The datasets used and/or analyzed during the current study are available from the corresponding author on reasonable request.

## Abbreviations

MNCAH: Maternal, newborn, child and adolescent health
MOH: Ministry of health
RBF: Result based financing
SARA: Service availability and readiness Assessment
WHO: World health organization
